# Study on the Impact of Income Gap on Health Level of Rural Residents in China

**DOI:** 10.3390/ijerph19137590

**Published:** 2022-06-21

**Authors:** Hongpeng Guo, Yang Yang, Chulin Pan, Shuang Xu, Nan Yan, Qingyong Lei

**Affiliations:** College of Biological and Agricultural Engineering, Jilin University, 5988 Renmin Street, Changchun 130022, China; ghp@jlu.edu.cn (H.G.); yangyang20@mails.jlu.edu.cn (Y.Y.); pancl@jlu.edu.cn (C.P.); xushuang_jlu@163.com (S.X.); yannan@jlu.edu.cn (N.Y.)

**Keywords:** health level, income gap, rural residents, probit model

## Abstract

With the rapid development of the social economy, health has increasingly become the focus of attention. Therefore, based on the balanced panel data of the China Household Tracking Survey (CFPS) from 2010 to 2018, the Probit model was used to investigate the impact of the income gap in rural areas on residents’ health level, and the relevant influencing mechanism was discussed in this paper. Results: (1) The income gap has a significant negative effect on the health level of rural residents, and the expansion of the income gap will have a more significant impact on the health level of rural residents. (2) The income gap will restrain the health level of rural residents by affecting the family income level and mobility constraints. (3) The restraining effect of the income gap on health formation mainly affects the families of young rural residents, rural male residents, residents with no rental income, and residents with low social capital. This paper analyzes and discusses, from the perspective of income gap, the impact of the income gap on the health status of rural residents in China. Based on the above conclusions, this paper puts forward some feasible suggestions to improve the health level of rural residents.

## 1. Introduction

Health is the foundation of people’s happiness and social development, the common pursuit of a better life by all Chinese people, and the basic requirement and an important support for achieving common prosperity. Since the reform and opening up, although China’s economy has maintained a high and steady growth, the income gap in rural areas has shown a widening trend. The widening of the income gap will lead to a series of economic and social problems and will also profoundly affect the health status of rural residents [1,2,3,4,5]. With the deployment of the important strategic decision of Healthy China, improving the income level of farmers, narrowing the income gap within rural areas, and maintaining the health of rural residents have become the strategic goals and the main direction of the whole of social development. Therefore, to study the impact of the income gap on the health level of rural residents is of great strategic significance for comprehensively improving the health level of residents and implementing the Healthy China strategy.

Residents’ health has attracted more and more attention. Many scholars have carried out studies on residents’ health [6,7] and have found that there is a correlation between income gap and health [8,9,10]. Some scholars believe that the expansion of the income gap will lead to an uneven allocation of resources, resulting in an insufficient supply of public medical facilities and an inadequate utilization of public medical services, thus adversely affecting health [11,12,13]. Some scholars believe that income will affect residents’ lifestyles, and low-income residents are more likely to form bad living habits, which will reduce their health status and thus affect their health [14,15]. Income also affects residents’ investment in education. Residents with a higher education level also have a higher health level. Studies have found that the impact of education on health is far greater than that of income [15,16]. In early studies, health was generally measured by mortality and life expectancy [9,17,18,19]. With the development of survey technology, more and more survey data contain detailed individual health information. Data at the microlevel has become the mainstream of research, and subjective health measurement is mostly adopted. The most common subjective health indicator is self-rated health [20,21,22].

At present, most scholars believe that the expansion of the income gap will have a significant negative impact on people’s health level [23,24,25,26,27,28]. Rodgers analyzed the population data of 56 countries and verified that the impact of the income gap on health was significantly negative with cross-sectional data [9]. Kuznets proposed an inverted U-shaped hypothesis concerning the income gap: with the continuous development of the economy, the income gap first shows a trend of widening, and then keeps shrinking [29]. Some scholars believe that the relationship between income and health is not significant [30,31,32]. Mellor and Milyo found no significant relationship between income gap indicators at the state and city levels derived from the marginal model and self-rated health [30]. Kawachi found that individual level factors, self-rated health and self-rated health status, were not closely related through the behavioral risk factor monitoring system [33,34]. Case investigated the relationship between income and health by using data collected from an informal town in South Africa, and found that there was no significant correlation [35]. Some scholars believe that there is a positive relationship between the income gap and health. K Judge and I. Patterson believed that the income gap helped to improve people’s health to a certain extent [36].

To sum up, the existing studies on the impact of the income gap on health mainly analyze data at macro- and microlevels, and most of the studies focus on the comparison of the health levels of all residents and the health of urban and rural residents. However, the research on the impact of rural residents’ income gap on health needs to be further studied. This paper analyzes from the perspective of income gap, discusses the impact of the income gap on the health status of rural residents in China, and puts forward feasible suggestions to improve the health level of rural residents.

## 2. Materials and Methods

### 2.1. Data Source and Description

This article uses data from the China Family Panel Studies (CFPS). The database is a biennial national, multidisciplinary social tracking survey project provided by the China Center for Social Science Surveys at Peking University with a national representative sample of village (neighborhood), family, family member follow-up surveys, and investigation, family, and community multilevel data. The focus is on the economic and noneconomic welfare of Chinese residents. Research topics include economic activity, educational outcomes, family relationships and family dynamics, population migration, and health. It is a national, large-scale, multidisciplinary social tracking survey project. The data sample covers households in 25 provinces, autonomous regions, and municipalities in China, excluding Tibet, Xinjiang, Qinghai, Inner Mongolia, Ningxia, Hainan and Hong Kong, Macao, and Taiwan. The CFPS collection method is rigorous, involving a wide range of data and high quality. It reflects the social and economic development of our country and the change of the health condition of its residents. It is of great representativeness and research value and provides a reliable data source for the academic research of this paper.

This article uses balanced panel data from the CFPS 2010–2018. This paper mainly studies the relationship between residents’ income and health status. In the adult questionnaire, there is a detailed investigation on the health status information of the samples, which is consistent with the research content of this paper. Based on the data matching of household and adult questionnaires according to the household head code, the data of five years were matched. In addition, the samples of urban household registration, the samples of those living in urban areas, the samples of “village resettlement”, and the samples with serious data missing values and outliers were deleted. After data processing and matching, the annual sample number of the panel data composed of eligible rural household samples was 3665 households, and the panel observation data totaled 18,325 rural households.

### 2.2. Variable Selection

The explained variable concerned in this paper is the health level of rural residents, while there are many methods to measure health in the previous literature. In order to effectively estimate the impact of the income gap on the health level of rural residents, this paper referred to Zhou Guangsu [37] and Mangyo et al. [38]. The choice of self-rated health in the CFPS survey reflects the health level of residents. Since self-rated health is a comprehensive health index, it is subjective to choose a single variable to measure the health level. Memory is also a reflection of mental health, so in this paper, memory was further selected as the explained variable to estimate the robustness test. To prove the impact of the income gap on the health level of rural residents, this paper is based on question P201 in the questionnaire: “How do you think of your health?” This question was constructed by three kinds of measures of self-reported health indicators. The first indicator is self-rated health 1, which is mainly rated from 1 to 5 on a scale from “unhealthy” to “very healthy”. The second indicator is self-rated health 2, which assigns “very healthy” and “very healthy” to “3”, “relatively healthy” to “2”, and “fair” and “unhealthy” to “1”. The third indicator is set as a dummy variable of “0–1”. If the self-rated health 1 is greater than 3, it is “1”; otherwise, it is “0”. Beyond that, memory is based on question Q501: “Can you remember the main things that happened to you in a week?”, “can completely remember”, and “can remember most” are assigned a value of “1”, while the rest of the answers are “0”.

The core explanatory variable of this paper is the income gap, and we refer to the research on the income gap by Lin Mello [39] and Zhou Guangsu et al. [37]. It mainly calculates the Gini coefficient of the same community, district, and county level to measure the degree of income inequality. However, many economic activities are not the same as living in towns due to habits such as “self-sufficiency” in rural households. Income is not an accurate measure of a family’s ability to draw on financial resources. By contrast, the consumption expenditure of rural residents can more accurately reflect the economic level and economic status of the family. Therefore, based on the total household consumption expenditure, this paper measures the Gini coefficient of expenditure at the community level and obtains the income gap 1 to measure the income gap. In addition, this paper further calculates the Gini coefficient of income at the district and county level to obtain the income gap 2. This calculates the Gini coefficient of expenditure at the district and county level to obtain the income gap so to investigate the impact of different income gap measures on the health of rural residents.

Referring to the literature and related theories, and according to the health demand model proposed by Grossman, individual health is affected by income, medical services, lifestyle, and individual endowment [40]. Accordingly, the control variables selected in this paper include the age of the head of the household, the gender of the head of the household, marital status, years of education, family size, the proportion of elderly population, the proportion of children population, whether they own property, whether they live in central and western China, whether they have hospitalization experience, whether they have a smoking habit, and whether they have a drinking habit. See Table 1 and Table 2 for details.

### 2.3. Model Design

In order to further investigate the impact of the income gap on the health level of rural residents, according to the setting, the explained variable in the model is the “health” of rural residents, which is a dummy variable. Therefore, Mcewen [21] and other references were used to select the Probit model for the benchmark empirical test. The constructed Probit model is as follows:

(1) $$Y_{ijc}=1\left(\alpha GINI_{jc}+\theta X_{ijc}+City_{c}+\delta_{ijc}>0\right)$$

where $\delta_{ijc}\sim N\left(0,\sigma^{2}\right)$, i represents the i rural household, j represents the j rural community, and c represents the c city, and $Y_{ijc}$ is the health status of the heads of i rural households located in the j rural community in the c city. A value of 1 means healthy and a value of 0 means unhealthy. The main variables were health and memory. $GINI_{ijc}$ is the income gap of the c city and j rural community, which is mainly measured by using the Gini coefficient of the expenditure at the community level. In the follow-up robustness test, income gap 2 and income gap 3 are used, where $X_{ijc}$ is the control variable. It mainly includes household head characteristics, household characteristics, urban characteristics, and household head living habits characteristics, etc. $City_{c}$ is a virtual variable at the city level, used to control the fixed effect at the city level, and $\delta_{ijc}$ is a random perturbation term.

According to the discrete sorting data type of the self-rated health level of rural residents, and the data type of health level 1 and health level 2, this paper further uses the ordered Probit model to study the impact of the income gap on the health level of rural residents, and constructs the specific model as follows:

(2) $$Health_{ijc}^{*}=\alpha +\beta GINI_{jc}+\gamma X_{ijc}+City_{c}+\varepsilon_{ijc}$$

(3) $$Health_{ijc}=\{\begin{array}{l} 1\;\;\;if\;\;\;\;Health_{ijc}^{*}\leq r_{1}\\ 2\;\;\;if\;\;\;\;r_{1}<Health_{ijc}^{*}\leq r_{2}\\ 3\;\;\;if\;\;\;\;r_{2}<Health_{ijc}^{*}\leq r_{3}\\ 4\;\;\;if\;\;\;\;r_{3}<Health_{ijc}^{*}\leq r_{4}\\ 5\;\;\;if\;\;\;\;r_{4}<Health_{ijc}^{*} \end{array}$$

where the $r_{1}<r_{2}<\cdots <r_{4}$ parameters are to be estimated, which is known as the cut-off point. When the distribution of the disturbance term conforms to the normal distribution, the model is the Oprobit model. In addition, this paper conducts clustering tests on all empirical cases at the city level to obtain the robust standard error of clustering at the city level, so as to obtain more accurate empirical regression estimation results.

Considering that, in the empirical process of using the Probit model and Oprobit model, although the fixed effects and clustering at the city level are controlled, bidirectional causality, omitted variables, and selective bias may still form potential endogenous problems. In particular, the model is affected by the problem of missing variables caused by the changes of the characteristics of the city’s sublevel and time dimensions. In order to overcome the endogenous problems caused by the above potential problems. This paper uses CFPS panel data for five years from 2010 to 2018 to control the missing variables at the city level that do not change over time through the fixed effect of the city and year. A bidirectional fixed effect model was used to investigate the impact of the income gap on the health of rural residents.

## 3. Results

### 3.1. Benchmark Empirical

Table 3 reports the empirical results of the Probit model on the impact of the income gap on the health level of rural residents. It shows the marginal effect of each variable and the clustering robust standard error of the corresponding city level in brackets. The explanatory variable was the health status of rural residents, and the core explanatory variable was the degree of the income gap at the community level. In the empirical process, the household head and the household characteristic variables, the regional variable characteristic variables, and the household head personal habits characteristic variables were controlled, respectively, and the fixed effect at the city level was controlled.

Among them, the empirical content in the first column only examines the effect of the core explanatory variables. The second column controls the fixed effects at the city level based on the first column. Each characteristic control variable is added to the third and fourth columns, respectively. As can be seen from the estimation results in column 4 of Table 3, the marginal effect of the Gini coefficient of community-level expenditure is −0.239, which is significant at the statistical level of 1%. That is, the expansion of the Gini coefficient has a significant negative impact on the health of rural residents. It shows that, with the widening of the income gap within the community, the health level of rural residents has decreased significantly. As can be seen from the empirical results from columns 1 to 4, regardless of whether other variables are controlled, the widening of the community income gap significantly inhibits the health of rural residents, which remains significant in all the empirical results.

### 3.2. Robustness Test

After performing the benchmark empirical regression analysis of investigating the income gap to rural resident’s health level. To further test the effectiveness of this effect, the robustness test was carried out from different indicators measuring explained variables and different indicators measuring explained variables.

#### 3.2.1. Different Explained Variables

First, this paper examines the impact of income inequality on different health indicators based on the Gini coefficient of expenditure measured at the community level. Health level 1, health level 2, and memory were used for the empirical tests so to test whether the impact of the income gap on the health level of rural residents changes with the change of health level of residents. The results of the regression empirical study by Oprobit are shown in Table 4.

In Table 4, health level 1 is used as the explanatory variable in columns 1 and 2, health level 2 is used as the explanatory variable in columns 3 and 4, and memory status is used as the explanatory variable in columns 5 and 6. According to Table 4, the effect of the income gap on the above variables is still significantly negative. Among them, the marginal effect of the income gap on health level 1 is −0.185, the marginal effect on health level 2 is −0.26, and the marginal effect on memory is −0.155, and this effect keeps a high significance level and effect. That is, the income gap has a significant inhibitory effect on all the indicators measuring the health level of rural residents. The above conclusions confirm the negative impact of the income gap on the health level of rural residents, and this effect remains significant under other models.

#### 3.2.2. Different Explanatory Variable 1: Income Gap (Measured by Income)

Secondly, this paper measures the income gap by measuring the Gini coefficient of income at the community level, providing further analysis of the impact of other indicators of the income gap on the health level of rural residents. This empirical result is reported in Table 5. The explained variables in columns 1 and 2 are the health status of rural residents, and the explained variables in columns 3 to 4 are health level 1 and health level 2, respectively.

As can be seen from the results in Table 5, after controlling for various control variables, other indicators of the income gap have a significant negative impact on the health level of rural residents. The marginal effects of income disparity in columns 2 to 4 are −0.275, −0.258, and −0.380, respectively. By comparing the difference of the marginal effect between the Gini coefficient of expenditure and the Gini coefficient of income, it can be found that the two have little difference in effect. Moreover, the income gap still has a significant inhibiting effect on the health of rural residents under the Gini coefficient of income. Therefore, it is reasonable to choose the Gini coefficient of expenditure to measure the income gap.

#### 3.2.3. Different Explanatory Variable 2: Income Gap (Measured by Consumption Expenditure)

Thirdly, this paper measures the income gap by measuring the Gini coefficient of the expenditure at the district and county level to investigate the impact of other measures of the income gap on the health level of rural residents.

Table 6 reports the empirical results, and the selection of the explained variables and model settings are consistent with Table 5. The empirical results in Table 6 show that, after controlling for various control variables, other indicators measuring the income gap have a significant negative impact on the health level of rural residents. The marginal effects of income disparity from columns 2 to 4 are −0.286, −0.308, and −0.394, respectively. That is, the effect of the income gap on the health level of rural residents has significantly expanded. It can be found that a more obvious degree of income gap can be measured by extending the calculation of the income gap at the level of the district and county. Moreover, the effect of this variable on the health level of rural residents has also been enhanced. It also shows that, with the increase of the income gap, the health level of rural residents will have a more obvious inhibiting effect.

### 3.3. Endogeneity Analysis

Although the impact of the income gap on the health level of rural residents has been verified in the benchmark empirical and robustness tests, the variables affecting the health level of rural residents were also controlled from family characteristics, household habits, and urban characteristics. However, problems such as bidirectional causality, omitted variables, and selective bias may still form potential endogenous frontal problems. In particular, it is affected by the problem of missing variables caused by the changes of the characteristics of the city’s sublevel and time dimensions. Therefore, in this paper, the health status of rural residents is regarded as a continuous variable, and a linear panel data bidirectional fixed effect model is used for the empirical regression estimation, both of which are controlled for the individual fixed effect at the city level and the time fixed effect at the year level. This empirical estimate is reported in Table 7.

In addition, the lower health status of people living in rural areas may lead to a lower household income and a lower consumption propensity. It may also lead to lower local income, and thus form a higher regional income gap. So, this can lead to a two-way causality problem. At the same time, the impact of family income status on residents’ health level also exists. Therefore, this paper still chooses the social capital of rural households as an instrumental variable. To investigate the impact of family income status on the health level of rural residents, from the perspective of rural households, the endogeneity problem can be alleviated. To comprehensively examine the impact of the income gap on the health level of rural residents, Table 8 reports the inspection results of the instrumental variable.

#### 3.3.1. Bidirectional Fixed Effects Model

In the empirical results of Table 7, the first listed is the result of the rural residents’ health, and the second and the third columns are health level 1 and health level 2, respectively. According to the empirical results, when the fixed effect of years is controlled, the income gap still has a significant negative impact on the health level of rural residents, and it is generally significant at the statistical level of 1%. By comparing the empirical results with Table 3, it can be found that the results of all columns in Table 7 remain basically robust. Although the marginal effect value of the impact of the income gap decreased, the impact on residents’ health remained above 6%, maintaining a stable effect. Therefore, when the bidirectional fixed effect model is used to alleviate endogenous problems, the effect of the income gap on the health level of rural residents still maintains an obvious inhibitory effect. It also shows the robustness of the empirical analysis.

#### 3.3.2. Instrumental Variable Method

This paper further discusses endogeneity based on balanced panel data and the instrumental variable method of the CFPS for five years from 2010 to 2018. The explanatory variable was the health status of rural residents, and the core explanatory variable was family income status. Family social capital was selected as the instrumental variable (logarithm), and the other control variables and the fixed effect control were consistent with Table 7.

According to the empirical estimation results of the least square method in Table 8, on the premise of meeting the weak instrumental variable test, choosing family social capital as the instrumental variable still has a significant positive impact on the health level of rural residents. The influence coefficients are generally significant at the statistical level of 1%, and this empirical result is basically consistent with Table 5. Therefore, the above empirical content has strongly verified that the income gap has a significant inhibitory effect on the health level of rural residents. Next, this paper will conduct an in-depth analysis on the heterogeneity of the influence mechanism and income gap.

### 3.4. Further Analysis

#### 3.4.1. Mechanism Analysis

The above empirical analysis has confirmed the negative impact of the income gap on the health level of rural residents. However, the above conclusions still do not discuss the way through which the income gap affects the health level of rural residents. The income gap will affect people’s relative status, and then also affect people’s material desires [41,42]. The income gap of residents’ health level inhibition may be through the income inhibition mechanism, leading to the family business, economic activity, and consumption of rural residents to be blocked [43].

To this end, reference is made to Zhou Guangsu [44]. In this paper, the household per capita income (logarithm) and liquidity constraint (household income with average monthly expenditure of more than three months = 1) were selected as the mechanism variables to discuss the influencing mechanism. Table 9 and Table 10 report the impact of the income gap on household income level and household mobility constraints.

According to the empirical results in Table 9, the widening of the income gap in the first column significantly restrains the per capita income level of households. That is, with the expansion of regional income gap, rural household income decreases. The empirical results from columns 2 to 4, respectively, report the impact of the income gap and the per capita income level on the health status, health level 1, and health level 2 of rural residents. It can be found that the increase of the per capita household income contributes to the improvement of rural residents’ health.

According to the empirical results of the income gap, family mobility constraints, and health level of rural residents in Table 10, in the first column of the empirical results, the widening of the income gap significantly increases the degree of the household mobility constraint. It shows that the possibility of rural households facing mobility constraints will increase by 5.3% when the income gap increases by one unit. That is, with the widening of the regional income gap, rural households face more severe mobility constraints, which is consistent with the findings of Yin Zhichao et al. [45]. The empirical results from columns 2 to 4, respectively, report the impact of the income gap and household mobility constraints on the health status, health level 1, and health level 2 of rural residents. It can be found that the deepening of household mobility constraints will inhibit the health level of rural residents.

#### 3.4.2. Heterogeneity Analysis

Next, this paper divides the households according to their age, gender, rental income, and social capital. Furthermore, it explores the group difference of the income gap’s effect on the health level of different rural households.

Table 11 reports the results of the heterogeneity of income disparities. First of all, columns 1 and 2 in Table 11 are divided according to the age of the head of the household. According to whether the head of the household is over 60 years old, it is divided into elderly households and nonelderly households. The results show that the restraining effect of the income gap formation is mainly on nonelderly household heads. The marginal effect value is −0.156, indicating that the income gap has a greater health inhibition effect on young rural residents. Secondly, columns 3 and 4 are divided into male-headed households and female-headed households according to the gender of the household head. The results show that the income gap has a significant negative impact on male rural residents.

Again, columns 1 and 2 of Table 12 are based on whether the rural household has rental income, including households with rental income and households without rental income. The marginal effects of the income gap on the health level of rural residents with or without rent income were 0.01 and −0.138, respectively. It can be found that the income gap has a more significant effect on the health level of rural residents without rental income. These results indicate that the additional source of property income in rural households can alleviate the inhibiting effect of the income gap on rural residents’ health level to a certain extent. Finally, columns 3 and 4 of Table 12 group rural households according to their social capital. The social capital is divided into high social capital families and low social capital families according to whether they exceed the average social capital. The results show that the restraining effect of the income gap formation mainly affects households with low social capital, and the marginal effect value is −0.194. This indicates that the income gap has a greater effect on the health of rural residents with low social capital.

## 4. Discussion

### 4.1. Discussion on the Impact of Income Gap on Health

From the conclusion of the study, the income gap significantly inhibits the health level of rural residents. This conclusion is consistent with the research conclusions of Tibber et al., Hurleya et al., and Bocoum et al. [46,47,48]. In addition, this paper focuses on identifying the effect of the income gap more accurately, and further provides a regression analysis by using the different explained variables, explanatory variables, and alternative measurement methods in the robustness test. In addition, a bidirectional fixed effect model and an instrumental variable method are used to solve the endogeneity problems. The above empirical attempts have obtained relatively robust research results, which are innovative in the use of the research methods, making the research conclusions more convincing and providing new empirical evidence and practical reference.

From the perspective of the research, this paper focuses on the mechanism behind the health damage caused by the income gap and reveals the impact and mechanism of the income gap on the health of rural residents from the perspective of health capital accumulation and opportunity inequality. It is found that the income gap inhibits the health of rural residents by influencing the family income level and restricting mobility. Possible reasons are that the rural household income level, family life level, level of consumer spending, and pay for healthcare ability is also improved, which also increases the health level of residents, but the expansion of the income gap easily restrains rural residents’ income increases, thus causing the deterioration of the residents’ health level. The possible reason is that the deepening degree of the mobility constraint will inhibit family economic activities and human capital investment activities, and will inhibit the improvement of the family income level of rural residents, thus resulting in the decline of residents’ health level.

In terms of the heterogeneity analysis, the group difference of the impact of the income gap on the health level of different rural households was further discussed. The study found that male heads of households had higher health levels than female rural residents. This conclusion is consistent with the research conclusions of Stefko et al., Roxo et al., and Bimpong et al. [49,50,51]. The reason for this phenomenon can be explained by “social causality theory”. Most men in rural areas are seriously patriarchal, while most women in rural areas need to do farm work, take charge of household chores, and undertake the double responsibilities of family production and family care. The salary of women is lower than that of men with the same education background and position, so women have always been in a disadvantaged position in social stratification. Due to their low social status, women’s access and ability to obtain health resources in the society are also very poor, thus affecting women’s health.

It is found that the health level of residents who own property is higher than that of residents who do not own property, which shows that owning property has a significant promoting effect on the health level of rural residents. This conclusion is consistent with the research conclusions of Hernandez and Swope and Narine and Shobe [52,53]. This phenomenon probably has something to do with the idea of “living and working in peace and contentment” since ancient times. At the same time, with the rapid rise of housing prices and land prices in China in recent years, the daily rent cost of migrant workers has become higher and higher, which has brought great economic and psychological burden to the residents renting houses [54].

It is found that the restraining effect of the income gap formation is on households with a low social capital. This conclusion is consistent with that of Karhina et al. and Akaeda [55,56]. The possible reason is that the income gap between residents will erode social capital to a certain extent. As the income gap between residents keeps widening, social capital is weakened, and public cohesion is reduced. Because of this, the social trust between people is greatly reduced, which will directly affect the physical and mental health of residents [57].

### 4.2. Policy Implications

Based on the above conclusions, in order to alleviate the restraining effect of the income gap on residents’ health level and improve residents’ health level, this paper puts forward policy suggestions, as follows:

(1) Improve the redistribution system, narrow the income gap, and improve the health of rural residents. The results of this study show that the income gap has a significant impact on health. Moreover, the greater the income gap, the more obvious the health inhibition effect on rural residents. The widening of the income gap will inevitably affect the development of social fairness and stability. The government should further improve the redistribution system by improving the tax system, providing tax relief policies for some people with economic difficulties or special circumstances, and improving the utilization efficiency of government funds. To solve the problem of the distribution gap is also important so to ensure rural residents’ income level is balanced.
(2) Improve the social security service system to alleviate the damage of the income gap to the health of vulnerable groups. As an important means of adjusting income distribution, social security is an important method and institutional guarantee to aid rural residents to share the fruits of reform and development, which can effectively improve the widening impact of the income gap in rural areas. Therefore, to promote the development of the social security service system in rural areas, we should emphasize the key points, to establish and improve the rural social security system through the basic medical security system, increase the tilt of medical resources in rural areas, and so on, to establish a multilevel social security system.
(3) Promote rural revitalization, increase employment opportunities, and improve the income gap for rural residents to avoid income restrictions. Increasing residents’ income is not only the basic guarantee of the rural revitalization strategy, but also the key to achieve rural revitalization. It is found that the increase of the per capita income of rural households contributes to the improvement of rural residents’ health. Therefore, it is necessary to enhance employment capacity and increase employment opportunities by strengthening policy incentives, building employment platforms, taking multiple measures to increase employment opportunities, and strengthening employment training. This will fundamentally solve the problem of income.
(4) Continuously enrich the financial infrastructure construction in rural areas to alleviate the liquidity constraint caused by the income gap. The income gap imposes mobility constraints on rural households and severely limits the ability and opportunities of family development. Inclusive finance will provide financial support for farmers’ development and more inclusive financial support for farmers’ entrepreneurship in order to help more families have the opportunity to develop, to increase the income of rural households, and thus help families get more needed medical resources and increase the optimal health capital stock.

## 5. Conclusions

Panel data of the CFPS from 2010 to 2018 are used in this paper. The Probit model was used to investigate the impact of the income gap on the health level of rural residents. The findings are:

First, income disparities significantly inhibit the health of rural residents. The results are still robust in the robustness test. It can be found that the inhibiting effect of the income gap on health is more strongly caused by the income gap in a limited range, while the effect of the income gap in a larger range is relatively weak. This strongly indicates that the continuous expansion of the regional income gap will inhibit the health level of rural residents and hinder the development of rural revitalization.

Secondly, the influencing mechanism is discussed. Studies show that the income gap inhibits the health of rural residents by influencing the household income level and restricting mobility. With the increase of rural residents’ household income, their living standards, consumption expenditure levels, and ability to pay for medical care have also improved, as has their health level. However, the expansion of the income gap tends to restrain the increase of rural residents’ income, which leads to the decline of residents’ health level. The deepening degree of the liquidity constraint will restrain household economic activities and human capital investment activities, and also exert a restraining influence on the improvement of the rural household income level. Therefore, the health level of residents declines.

Finally, the heterogeneity was analyzed to further explore the group difference of the income gap’s effect on the health level of different rural households. The results show that the restraining effect of the income gap formation mainly affects nonelderly household heads, rural male residents, households with low social capital, and rural residents without rental income.

## Figures and Tables

**Table 1:** Variable definitions.

Variable	Definition
Core explanatory variable	
Income gap	Total household consumption expenditure measures by the Gini coefficient of spending at the community level
Control variables	
Age	Respondents age
Gender	Men = 1; Women = 0
Marital status	Married = 1; Unmarried = 0
Years of education	High school degree or above = 1; Education below high school = 0
Family size	The number of family members
Proportion of elderly population	Percentage of households aged 65 and over
Proportion of children in the population	Percentage of households under the age of 18
Property ownership	Owning one or more houses = 1; No property = 0
Midwest residence	The midwest = 1; Other regions = 0
Hospitalization	Hospitalization due to illness in the past 12 months = 1; Not in the hospital = 0
Smoking	Smoking = 1; No smoking = 0
Drinking	Drinking = 1; No alcohol = 0

**Table 2:** Descriptive statistics.

Variable	Observation	Mean Value	Standard Deviation	Minimum Value	Maximum Value
Health	18,325	0.680	0.467	0.000	1.000
Health Level 1	18,325	3.104	1.322	1.000	5.000
Health Level 2	18,325	2.069	0.840	1.000	3.000
Memorization	18,325	0.423	0.494	0.000	1.000
Income gap 1	18,325	0.417	0.081	0.114	0.775
Income gap 2	18,325	0.448	0.057	0.261	0.727
Age	18,325	55.133	13.247	20.000	90.000
Gender	18,325	0.792	0.406	0.000	1.000
Marital status	18,325	0.917	0.276	0.000	1.000
Years of education	18,325	6.174	4.019	0.000	16.000
Family size	18,325	4.157	1.821	1.000	21.000
Proportion of elderly population	18,325	0.155	0.264	0.000	1.000
Child population ratio	18,325	0.274	0.230	0.000	0.900
Own property	18,325	0.923	0.266	0.000	1.000
Central and western Regions	18,325	0.606	0.489	0.000	1.000
Experience in hospital	18,325	0.087	0.282	0.000	1.000
Smoking habits	18,325	0.493	0.500	0.000	1.000
Drinking habits	18,325	0.273	0.445	0.000	1.000

**Table 3:** Impact of income gap on health of rural residents.

Variable	(1)	(2)	(3)	(4)
	Probit	Probit	Probit	Probit
	Health	Health	Health	Health
Income gap	−0.272 ***	−0.276 ***	−0.244 ***	−0.239 ***
	−0.068	−0.066	−0.061	−0.059
Age			−0.005 ***	−0.005 ***
			0	0
Gender			0.116 ***	0.069 ***
			−0.013	−0.014
Marital status			0.033 *	0.029 *
			−0.018	−0.017
Years of education			0.010 ***	0.010 ***
			−0.001	−0.001
Family size			0.003	0.002
			−0.003	−0.002
Proportion of elderly population			0.042 **	0.043 **
			−0.021	−0.02
Child population ratio			0.103 ***	0.096 ***
			−0.022	−0.022
Own property			0.028 *	0.027 *
			−0.016	−0.015
Central and western regions			−0.121	−0.1
			−0.116	−0.117
Experience in hospital				−0.219 ***
				−0.011
Smoking habits				−0.037 ***
				−0.009
Drinking habit				−0.069 ***
				−0.01
Urban fixed effect		Control	Control	Control
Observed value	18,325	18,325	18,325	18,325

Note: The empirical results reported in this paper only calculate the marginal effect of core explanatory variables, and the marginal effect results of other control variables are not reported. The brackets are clustering robust standard errors at the district/county level, where ***, **, and * represent *p* values less than 0.01, 0.05, and 0.1, respectively.

**Table 4:** The impact of income gap on different health indicators.

Variable	(1)	(2)	(3)	(4)	(5)	(6)
	Oprobit	Oprobit	Oprobit	Oprobit	Probit	Probit
	Health Level 1	Health Level 1	Health Level 2	Health Level 2	Memorization	Memorization
Income gap	−0.212 ***	−0.185 ***	−0.297 ***	−0.260 ***	0.171 **	−0.155 **
	−0.053	−0.047	−0.075	−0.067	−0.07	−0.069
Control variables	Control	Control	Control	Control	Control	Control
Urban fixed effect	Control	Control	Control	Control	Control	Control
Observed value	18,325	18,325	18,325	18,325	18,325	18,325

Note: *** and ** represent *p* values less than 0.01 and 0.05, respectively.

**Table 5:** Impact of different income gap indicators on health level of rural residents (Measured by Income).

Variable	(1)	(2)	(3)	(4)
	Probit	Probit	Oprobit	Oprobit
	Health	Health	Health Level	Health Level
Income gap	−0.336 ***	−0.275 ***	−0.258 ***	−0.380 ***
	−0.048	−0.042	−0.032	−0.047
Control variables	Control	Control	Control	Control
Urban fixed effect	Control	Control	Control	Control
Observed value	18,325	18,325	18,325	18,325

Note: *** represents *p* value less than 0.01.

**Table 6:** Impact of different income gap indicators on health level of rural residents (Measured by Consumption Expenditure).

Variable	(1)	(2)	(3)	(4)
	Probit	Probit	Oprobit	Oprobit
	Health	Health	Health Level 1	Health Level 2
Income gap	−0.397 ***	−0.286 **	−0.308 ***	−0.394 ***
	(0.124)	(0.116)	(0.102)	(0.146)
Control variables	Control	Control	Control	Control
Urban fixed effect	Control	Control	Control	Control
Observations	18,325	18,325	18,325	18,325

Note: *** and ** represent *p* values less than 0.01 and 0.05, respectively.

**Table 7:** Empirical estimation results of bidirectional fixed effects model.

Variable	(1)	(2)	(3)
	Probit	Oprobit	Oprobit
	Health	Health Level 1	Health Level 2
Income gap	−0.130 ***	−0.069 ***	−0.089 **
	(0.049)	(0.026)	(0.043)
Age	−0.004 ***	−0.003 ***	−0.004 ***
	(0.000)	(0.000)	(0.000)
Gender	0.068 ***	0.056 ***	0.083 ***
	(0.014)	(0.009)	(0.013)
Marital status	0.027	0.021 **	0.031 **
	(0.017)	(0.010)	(0.015)
Years of education	0.010 ***	0.004 ***	0.005 ***
	(0.001)	(0.001)	(0.001)
Family size	0.006 ***	0.005 ***	0.006 ***
	(0.002)	(0.002)	(0.002)
Proportion of elderly population	0.030	0.016	0.026
	(0.020)	(0.011)	(0.017)
Child population ratio	0.011	0.010	0.010
	(0.020)	(0.012)	(0.012)
Own property	0.030 **	0.015 *	0.024 *
	(0.014)	(0.008)	(0.012)
Central and western regions	−0.122	−0.026	−0.072
	(0.106)	(0.051)	(0.078)
Experience in hospital	−0.215 ***	−0.158 ***	−0.216 ***
	(0.011)	(0.007)	(0.012)
Smoking habits	−0.029 ***	−0.018 ***	−0.022 ***
	(0.009)	(0.005)	(0.008)
Drinking habits	−0.070 ***	−0.039 ***	−0.053 ***
	(0.010)	(0.006)	(0.009)
Urban fixed effect	Control	Control	Control
Year fixed effect	Control	Control	Control
Observations	18,325	18,325	18,325

Note: ***, **, and * represent *p* values less than 0.01, 0.05, and 0.1, respectively.

**Table 8:** Empirical estimation results of least square method.

Variable	(1)	(2)	(3)
	2SLS	2SLS	2SLS
	Health	Health	Health
Family income status	0.050 ***	0.086 ***	0.037 **
	(0.014)	(0.014)	(0.016)
Control variables	Control	Control	Control
Constant term	0.535 ***	0.629 ***	0.717 ***
	(0.072)	(0.069)	(0.081)
Urban fixed effect	Control	Control	Control
Year fixed effect		Control	Control
Observations	18,325	18,325	18,325
*R* ^2^	0.045	0.072	0.152
Cragg–Donald Wald F	573.839	553.191	408.931
First class F	75.57	49.23	46.61

Note: *** and ** represent *p* values less than 0.01 and 0.05, respectively.

**Table 9:** Income gap, household income level, and health level of rural residents.

Variable	(1)	(2)	(3)	(4)
	OLS	Probit	Oprobit	Oprobit
	Per Capita Income	Health	Health Level 1	Health Level 2
The income gap	−0.519 **	−0.125 **	−0.066 **	−0.084 *
	(0.205)	(0.049)	(0.026)	(0.043)
Per capita household income		0.010 ***	0.007 ***	0.009 ***
		(0.002)	(0.001)	(0.002)
Constant term	8.599 ***			
	(0.151)			
Control variables	Control	Control	Control	Control
Urban fixed effect	Control	Control	Control	Control
Year fixed effect	Control	Control	Control	Control
*R* ^2^	0.150			
Observations	18,325	18,325	18,325	18,325

Note: ***, **, and * represent *p* values less than 0.01, 0.05, and 0.1, respectively.

**Table 10:** Income gap, mobility constraints, and health of rural residents.

Variable	(1)	(2)	(3)	(4)
	OLS	Probit	Oprobit	Oprobit
	Liquidity Constraint	Health	Health Level 1	Health Level 2
Income gap	0.053 ***	−0.122 **	−0.064 **	−0.081 *
	(0.002)	(0.049)	(0.026)	(0.043)
Liquidity constraint		−0.047 ***	−0.029 ***	−0.043 ***
		(0.007)	(0.004)	(0.006)
Control variables	Control	Control	Control	Control
Constant term	−1.259 ***			
	(0.118)			
Urban fixed effect	Control	Control	Control	Control
Year fixed effect	Control	Control	Control	Control
*R* ^2^	0.231			
Observations	18,325	18,325	18,325	18,325

Note: ***, **, and * represent *p* values less than 0.01, 0.05, and 0.1, respectively.

**Table 11:** Heterogeneity analysis results (age and gender differences).

Variable	(1)	(2)	(3)	(4)
	Elderly Head of Household	Nonelderly Head of Household	Male Head of the Household	Female Head of the Household
	Probit	Probit	Probit	Probit
	Health	Health	Health	Health
Income gap	−0.088	−0.156 ***	−0.144 ***	−0.039
	(0.085)	(0.059)	(0.054)	(0.095)
Control variables	Control	Control	Control	Control
Urban fixed effect	Control	Control	Control	Control
Year fixed effect	Control	Control	Control	Control
Observations	6403	11,922	14,505	3820

Note: *** represents *p* value less than 0.01.

**Table 12:** Heterogeneity analysis results (whether there are differences between rental income and social capital).

Variable	(1)	(2)	(3)	(4)
	Rental Income	Nonrental Income	High Social Capital	Low Social Capital
	Probit	Probit	Probit	Probit
	Health	Health	Health	Health
Income gap	0.010	−0.138 ***	−0.095 ***	−0.194 ***
	(0.167)	(0.050)	(0.006)	(0.037)
Control variables	Control	Control	Control	Control
Urban fixed effect	Control	Control	Control	Control
Year fixed effect	Control	Control	Control	Control
Observations	1606	16,719	6002	12,323

Note: *** represents *p* value less than 0.01.

## Data Availability

Not applicable.
